# PROMOTING PARTICIPATION IN OCCUPATIONS AMONG OLDER ADULTS LIVING WITH NEUROCOGNITIVE DISORDERS AND CAREGIVERS

**DOI:** 10.1093/geroni/igad104.2757

**Published:** 2023-12-21

**Authors:** Patricia Belchior, Melanie Levasseur, Johanne Filiatrault, Meena Ramachandran, Nathalie Bier

**Affiliations:** McGill University, Montreal, Quebec, Canada; Université de Sherbrooke, Sherbrooke, Quebec, Canada; Université de Montréal, Montreal, Quebec, Canada; McGill University, Montreal, Quebec, Canada; Université de Montréal, Montréal, Quebec, Canada

## Abstract

Participation in meaningful occupations promotes self-confidence, self-esteem, overall health, and healthier life transitions. Due to significant life changes brought by neurocognitive disorders older adults living with neurocognitive disorders as well as caregivers may undergo some disruption in their occupational participation. Few interventions have focused on their ability to engage in meaningful occupations independently and together to successfully orchestrate their life to meet their own occupational needs. The goal of this study was to document the priority needs of older adults living with neurocognitive disorders and caregivers to promote occupational participation, to guide us in the development of an intervention. To this end, a rapid literature review was conducted. Three databases (Medline, PsycInfo and CINAHL) were searched. Studies were included if they used qualitative methods, included community-based individuals and were available in English or French. A total of 20 studies met the inclusion criteria. Two needs were prioritized in the literature by both older adults living with neurocognitive disorders and caregivers: 1) to continue participating in daily activities and social roles (e.g., maintain the connection with the community, use everyday technology), 2) to educate the public about the disease (e.g., bring awareness, knowledge, and understanding of dementia among the community at large). In the presentation we will discuss how the results will inform the development of an occupational-based intervention to promote meaningful occupations.

